# A unique approach for investigating skin lesions and their relationship with TCM syndrome differentiation in alopecia areata

**DOI:** 10.4314/ahs.v24i3.45

**Published:** 2024-09

**Authors:** Xianzhe Zhang, Man Zhang, Wen Guo, Jianhui Guo

**Affiliations:** 1 Department of Traditional Chinese Surgery, Hebei University of Chinese Medicine, Shijiazhuang 050200, China; 2 Cangzhou Hospital of Integrated TCM-WM · Hebei, Cangzhou 061000, China

**Keywords:** Dermatoscopic signs, Alopecia areata, TCM syndromes, Qi and blood deficiency, Liver and kidney deficiency

## Abstract

**Background:**

Alopecia areata is also known as ‘oil wind’ in Chinese medicine. Alopecia areata is a debilitating autoimmune skin disease characterized by patches of non-scarring hair loss and inflammation that damages the scalp and body hair in a variable, often relapsing or permanent fashion. It is not quite obvious how the various Traditional Chinese Medicine (TCM) syndromes differentiate between the skin lesions under dermatoscopy caused by alopecia areata.

**Objective:**

This study aimed to provide a rational basis for TCM syndrome differentiation of alopecia areata and investigate the role of skin lesions in various TCM syndromes of alopecia areata under dermatoscopy.

**Methods:**

The dermatologist used a dermatoscope to take four images of the patient's skin lesion area and the skin lesion junction. Two of the images were captured with polarized light, while the other two were taken with non-polarized light. Patients with alopecia areata who had TCM syndrome differentiation were identified and examined using dermatoscopy. The occurrence of the yellow dot, white dot, exclamation mark hair and vascular sign, was determined and then statistically analyzed.

**Results:**

The positive rates of vascular signs relate to alopecia areata, exclamation mark hair relates to Qi-blood deficiency and white dot corresponded to liver and kidney deficiency. All the positive rates were statistically significant (P < 0.05). Compared to vascular sign, white dot sign, and Qi-blood deficiency syndrome groups, the positive rate of yellow dot syndrome was not significant (P>0.05)

**Conclusion:**

The findings suggest that particular skin lesions contribute to differentiating different TCM syndromes associated with alopecia areata. Skin lesions examined under dermatoscopy can assist in diagnosing the TCM syndromes associated with alopecia areata.

## Introduction

Alopecia areata is an autoimmune disorder that causes non-scarring hair loss. It often manifests as spherical patches of hair loss that are demarcated and can occur at any age. Alopecia areata is also known as “oil wind” in Chinese medicine. Clinically, there are numerous types of hair loss, ranging from localized patches (alopecia totalis,) to scattered or total hair loss on the body (alopecia universalize) without any symptoms. 1.

A comprehensive analysis of alopecia areata epidemiology found a 2% lifetime prevalence worldwide. Some smaller studies show a female-to-male gender bias; however, this may be related to female concern about hair loss and treatment. The lifetime incidence of the disease appears to rise exponentially. Additionally, the disease's progression is unpredictable, with 80% of patients experiencing spontaneous hair growth within the first year and the possibility of a rapid recurrence at any time. In a few patients, the disease may be prolonged for years or even decades 1.

The diseases' specific etiology is complex and currently unknown. Evidence suggests; However, that alopecia areata is caused by an autoimmune reaction to the hair follicles that are triggered by both genetic (Human leukocyte antigen-DR on chromosome) and environmental factors such as stress. Minoxidil tincture is regarded as a hair growth stimulation agent, clinically frequently used for the treatment of male alopecia and alopecia areata, although its efficacy for some patients is limited, and is easy to relapse after discontinuation 2.

Glucocorticoids are commonly used to treat alopecia areata, however, with several side effects like skin atrophy, hair folliculitis, hypopigmentation, and glaucoma 3.

Immunosuppressive agents are effective in some patients, but they are not used as first-line drugs because of their relatively large number of adverse reactions, relatively high cost and high recurrence rate after drug withdrawal. The application of Minoxidil is limited and must be used twice per day regularly and it only works if the medication remains on the scalp for between 3-4 hours. The Chinese medicine treatment for alopecia areata has a substantial clinical impact with minor side effects. According to the characteristics of skin lesions and accompanying symptoms, alopecia areata can be divided into blood heat syndrome, Qi stagnation and blood stasis syndrome, Qi blood deficiency syndrome, and liver and kidney deficiency syndrome 4.

Combining traditional Chinese and western medicine, including using current medical diagnosis and treatment procedures based on syndrome differentiation, increases the objectivity and accuracy of Traditional Chinese Medicine (TCM) syndrome differentiation.

Dermatoscopy is a unique, non-invasive diagnostic approach that can be used as a valuable tool for improving the accuracy of identifying common hair and scalp diseases and skin diseases. It was initially used for the diagnosis, treatment evaluation and treatment endpoint determination of melanoma. Currently, it is widely used to assist in the differential diagnosis and severity assessment of inflammatory diseases, hair diseases, skin infections, and nail diseases, etc. 5,6.

The polarized light mode of dermoscopy can improve the contrast of dermal tissue observation, while the non-polarized light mode has shallow tissue level. These two methods can complement each other. Dermatoscopy has steadily established a major role in diagnosing, assessing severity, and prognostic evaluation of alopecia areata 7,8. Bald spot commonly includes yellow dot sign, blood vessel sign, exclamation mark hair, and white dot sign. There are currently no studies to establish whether these signs are related to TCM syndrome types or whether they can help in TCM syndrome differentiation. 9-11. Combining dermatoscopic indicators with TCM syndrome differentiation can extend the depth of syndrome differentiation and improve accuracy.

In this study, the dermatoscopic signs of alopecia areata patients with different TCM syndromes were evaluated. The correlation between the disease conditions and TCM syndromes was analyzed to investigate the possibility of incorporating contemporary diagnostic and treatment modalities into the dialectical framework of TCM and offer a more holistic and objective point of view on the differentiation of TCM syndromes.

## Data and methods

### Subjects

This study was approved by Cangzhou Hospital of Integrated Traditional Chinese and Western Medicine, with written informed consent obtained from all participants. The study included one hundred twenty alopecia areata patients treated in the dermatology department of Cangzhou Hospital of Integrated Traditional Chinese and Western Medicine from November 2020 to November 2021. Patients were divided into 3 groups according to TCM syndrome type. There was no statistical significance in gender, age and other general data between groups (P > 0.05), indicating comparability. The study was conducted following western medicine diagnostic criteria “Chinese Alopecia areata Diagnosis and Treatment Guidelines (2019)” 3.

Patchy hair loss is usually round or oval in different sizes, which can be single or multiple, mainly occurring in heads, faces or bodies. Alopecia patches typically have clear boundaries and normal skin appearance without obvious self-conscious symptoms. A few patients may have mild scalp itching or scalp tightness.

The inclusion and exclusion criteria were followed.

### Inclusion criteria

1) Conform to the diagnostic criteria of alopecia areata;
2) Age range: 18-60;
3) Without any treatment, including oral, topical drugs and physical therapy;
4) Informed consent;
5) Follow the doctor's advice and be able to cooperate with the treatment actively;
6) The area of hair loss ≤ 30% of the area of the scalp: no internal, topical, or other related treatments related to the treatment of alopecia areata were used for 2 weeks before treatment.

### Exclusion criteria

1) 2 TCM physicians failed to achieve consistent syndrome differentiation or patients with concurrent syndrome differentiation;
2) Patients with unclear images collected by dermatoscopy;
3) patients with severe immune system diseases and endocrine diseases;
4) Pregnant and lactating women;
5) Patients with incomplete clinical information;
6) People with other scalp disorders such as congenital alopecia, pseudo-alopecia, seborrheic alopecia, syphilitic alopecia, lupus alopecia, and other drug-induced alopecia;
7) Patients treated with glucocorticoids and immunosuppressant systematically within one month before treatment.

Among the 120 patients, 65 were male and 55 were female. The age ranged from 18 to 51 years, with an average of 43.2±4.9 years. There were 40 patients in the blood hot air dryness group, 40 in the qi and blood deficiency group, and 40 in the liver and kidney deficiency group.

### Standard of TCM Syndrome differentiation

The standard of TCM syndrome differentiation reference from <Dermatology and Venereology of Traditional Chinese Medicine>; syndrome differentiation is performed by two or more two TCM physicians, respectively; and the outcomes of syndrome differentiation are all considered to be effective 4

The following criteria should be adopted for the differentiation;

1) The skin damage characteristics of blood hot air dryness syndrome: Sudden hair loss in pieces, occasionally itching scalp, or accompanied by the hot head.
  Accompanying symptoms: Often upset, irritability, impatient restlessness, red tongue, thin moss, and pulse string.
2) Skin lesions of qi and blood deficiency syndrome: the majority of the hair, following the disease or postpartum flake shedding, and gradual aggravation, from little to large, hair withered and thinning.
  Accompanying symptoms: white lips, palpitations, shortness of breath, lazy speech, fatigue, light tongue, thin white tongue coating, and weak pulse.
3) The skin lesions of liver and kidney deficiency syndrome: The disease has a prolonged progression duration. When the first signs of significant uniform shedding, or even hair loss, appear, the color of the hair is typically brown or grey.
  Accompanying symptoms: dizziness, tinnitus, vertigo, soft waist and knees, weak tongue, thin moss, and thin pulse.

### Dermatoscopy equipment for patient's inspection

The Dermatoscopy (BN-PFMF-8001, Nanjing Beining Medical Instrument Co., LTD) equipped with LED (illuminance ≥2000 Lux, imaging resolution ≥2592×1944, pixel ≥5 million) was used for skin and hair observation. Before the examination, all patients rested for three minutes to ensure that scalp temperature was not altered by physical activity. If the patient underwent strenuous exercise, drug bath and other activities to change the lesion status before the image collection, the patient should be stationary for 1 hour before the image collection. The physician took two polarized images and two non-polarized images of the patient's skin lesion region and skin lesion junction using a dermatoscope. Image quality control: When shooting, make the lens edge close to the skin, adjust the best Angle and image clarity, and retain the image after the completion of focusing.

### Observation indicators

**Figure 1:**
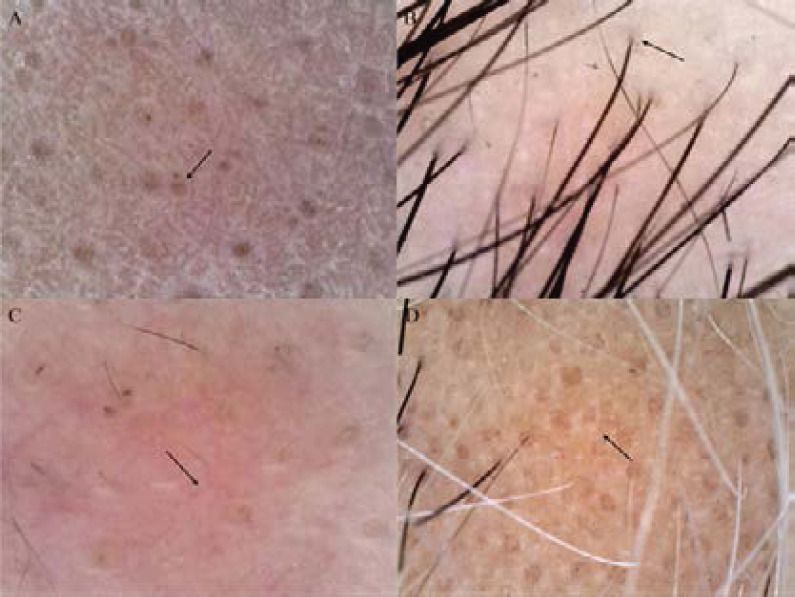
Observation indicators. A. Yellow dot sign; B. Exclamation mark hair; C. Vascular signs; D. White dot sign

The outcome measures were determined by two dermatologists with reference to the relevant definitions. Positive indicators were recorded if images showed corresponding signs. The four indicators are defined as follows: 1) Yellow spot sign: round or multi-ring yellow to yellow-pink spots at the opening of hair follicle unit (Figure 1 A).

Exclamation mark hair: refers to the thinning of the hair on the skin end, such as the thick hair on the exclamation mark (Figure 1 B),

Vascular signs: localized multiform capillaries under the skin (Figure 1 C).

The white spot sign is pin-sized white spots scattered between hair follicles (Figure 1 D).

### Statistical methods

SPSS 23.0 statistical software was used to analyze the data. Data was expressed as a percentage and statistical analysis was performed by χ2 test. P < 0.05 was considered statistically significant.

## Results

From the findings, we observed that the rate of positive vascular signs was higher (P<0.05) in the blood drying group than in the other two groups (Table 1). Similarly, we observed higher (P < 0.05) positive signs of the exclamation mark in the qi-blood deficiency group than other two groups (Table 2). Also, last but not least, the rate of positive white spot signs was higher (P<0.05) in the liver and kidney deficiency group compared to the other two groups (Table 3). However, no significant difference was observed in the positive rate of the yellow spot in the three groups (Table 4, P > 0.05).

**Table 1:** Comparison of vascular signs among the three groups

Group	Vascular sign (+)	Vascular sign (-)	Positive rate (%)
Blood hot air dryness group (n=40)	20	20	50.0 *#
Qi-blood deficiency group (n=40)	7	33	17.5
Liver and kidney deficiency group (n=40)	9	31	22.5

Note: +: Positive; -: negative. Compared with Qi-blood deficiency group, *P < 0.05; Compared with the liver and kidney deficiency group, #*P* < 0.05

**Table 2:** Comparison of exclamation marks among the three groups

Group	Exclamation mark hair (+)	Exclamation mark hair (-)	Positive rate (%)
Blood hot air dryness group (n=40)	12	28	30.0
Qi-blood deficiency group (n=40)	29	11	72.5*#
Liver and kidney deficiency group (n=40)	18	22	45.0

Note: Compared with the blood hot air dryness group, *P < 0.05; Compared with the liver and kidney deficiency group, #*P* < 0.05

**Table 3:** Comparison of white spot signs among the three groups

Group	Yellow dot sign (+)	Yellow dot sign (-)	Positive rate (%)
Blood hot air dryness group (n=40)	5	35	12.5
Qi-blood deficiency group (n=40)	6	34	15.0
Liver and kidney deficiency group (n=40)	15	25	37.5*#

Note: Compared with the blood hot air dryness group, *P < 0.05; Compared with Qi- blood deficiency group, #*P* < 0.05

**Table 4:** Comparison of yellow spot signs among the three groups

Group	Yellow dot sign (+)	Yellow dot sign (-)	Positive rate (%)
Blood hot air dryness group (n=40)	22	18	55.0
Qi-blood deficiency group (n=40)	30	10	75.0
Liver and kidney deficiency group (n=40)	26	14	65.0

Note: Not significant difference was observed among the three groups, *P* > 0.05

## Discussion

Alopecia areata is a common clinical disease in dermatology. Typical alopecia areata can be diagnosed on the basis of clinical presentation and dermoscopy without special examination. Dermoscopy served as a diagnostic method for alopecia areata and distinguished the condition from other hair loss conditions that could comparably manifest themselves. Dermoscopy a non-invasive microscopic image analysis technique that examines the structure down to the dermal papilla layer, which helps in understanding the characteristics of lesions in a complete and three-dimensional way. In recent years, this method has seen widespread application in clinical settings and has offered a whole new academic category. It possesses significant diagnostic, identifying, and evaluative versatility for various dermatological diseases, particularly hairy and pigmented skin diseases, and has become widely attractive 12,13.

Alopecia areata has more sensitivity and specificity signs such as yellow dots, black dots, broken hairs, exclamation mark hairs and short vellus hairs are significant indicating early regrowth. Yellow dots are the most prevalent and sensitive characteristic of alopecia areata. The yellow dot sign is the multi-ring structure formed by the accumulation of oil and keratin tissue at the funnel of the hair follicle. These dots represent the follicular infundibulum, distended with degenerating keratinocytes and sebum. Vascular signs are caused by capillary hyperemia and dilation, including point-shaped, globular, linear, etc., and the characteristics of the arrangement can be seen as branching and reticular, etc. The vascular signs are easily visible in those with fair skin, but in people with a darker complexion, they are obscured by a more prominent pigment pattern. The hair shaft of exclamation mark hair is thinner near the scalp formed by malnutrition under the attack of inflammatory cells in the hair follicle during growth. A white spot sign is a dense white spot caused by local loss of melanin after hair follicle destruction. White dots represent damaged follicles replaced by fibrous tracts and are more visible in dark-skinned people or over browned parts in fair-skinned people. 9-11, 14. Dermoscopy can improve the accuracy of diagnosis, which plays an important role in diagnosing and treating alopecia areata. TCM syndrome is a profile of symptoms and signs as a series of disease manifestations. Blood hot air dryness, qi and blood deficiency, and liver and kidney deficiency are the three common syndromes of this disease.

In this study, the patients with alopecia areata were observed with dermoscopy. The positive rates of yellow dot signs, vascular signs, exclamation mark hair and white dot signs were investigated. Correlations were observed between the blood hot wind dryness group and the vascular sign, the qi and blood deficiency group and the exclamation mark hair, the liver and kidney deficiency group and the white spot sign. The positive rate of yellow spot signs in the three groups was not statistically significant (Table 1-4). There was no statistical significance in the distribution of the yellow dot sign in the three groups.

Pathology of alopecia areata is characterized by mast cells infiltrating around superficial vessels of the dermis, releasing histamine to dilate arterioles and congestion 14. The formation of exclamation point hair is related to the destruction of hair follicle immunity. The release of interferon (IFN)-γ and tumor necrosis factor (TNF)-α induced by various factors leads to the abnormal expression of histocompatibility complex (MHC) class I molecules in a hair follicle during the growth period which destroys the immune immunity state of the hair follicle. At the same time, the hair follicles are attacked by inflammatory cells, and the hair near the scalp end becomes thinner and lighter in color 15, 16.

A white spot sign is a kind of sebaceous gland fibrosis or sweat gland structure, which mostly appears in the late stage of chronic hair disease 17, 18.

Considering its high sensitivity to alopecia areata, its dialectical significance can be further explored in the future^19^,^20^.

Syndrome differentiation and treatment reflect the ability of comprehensive understanding and treatment of individuals of disease, which is the treasure of Chinese medicine. On the other hand, due to the subjective nature of TCM's four diagnostic categories, there is sometimes lack of consistency in the syndrome distinction of the same object.

The four TCM syndrome diagnostic approaches should be developed further and expanded in light of advancements in contemporary diagnostic and therapeutic techniques, particularly at the micro-level. This study investigated the differences in skin signs in alopecia areata patients with different syndromes by analyzing the correlations between TCM syndromes and conventional diagnosis. It also offers the notion of syndrome differentiation for treating and diagnosing alopecia areata by combining TCM and dermatoscopy.

Nevertheless, this study has a few drawbacks, the most significant being the limited size of the study's sample population. The fact that the conjugation could not be investigated during this investigation is another factor that unquestionably restricts how far the findings of this study can be extrapolated. It is anticipated that additional clinical studies with large samples, many centers and several lines of evidence will be required. Future research on the association between dermatoscopic signs and TCM syndrome differentiation can improve the content of TCM syndrome differentiation and promote the development of TCM and WESTERN medicine integration.

## Conclusion

The skin lesions under dermatoscopy of alopecia areata patients correlate with their TCM syndrome differentiation.

Clinical Trial Registration Number: ChiCTR-POC-17002561
